# Comparison of Radiographic and Ultrasound Imaging Techniques for Assessing the Scapholunate Interval in Healthy Individuals

**DOI:** 10.3390/jcm15114250

**Published:** 2026-05-31

**Authors:** Grégoire Micicoi, Jean Baptiste De Villeneuve Bargemon, Pharel Njessi, Thomas Apard, Nicolas Dréant, Elise Lupon

**Affiliations:** 1 Institut Universitaire Locomoteur et du Sport, Pasteur 2 Hospital, Université Côte d’Azur, 06001 Nice, France; 2 Hand Surgery and Limb Reconstructive Surgery Department, La Timone Adult Hospital, Aix Marseille University, 13005 Marseille, France; 3 Laboratory of Molecular PhysioMedicine (LP2M), UMR 7370, CNRS, Université Côte d’Azur, 06000 Nice, France; 4 Ultrasound Hand Surgery Center, 2 Rue de Tocqueville, 78000 Versailles, France; 5 Clinique Saint François, 10 Boulevard Pasteur, 06000 Nice, France

**Keywords:** ultrasound, scapholunate instability, radiographic stress view, wrist ultrasonic examination, wrist examination, scapholunate gap, scapholunate noninvasive assessment

## Abstract

**Background/Objectives:** Ultrasound (US) is a valuable tool in hand surgery because of its portability, low cost, and ability to provide noninvasive, real-time evaluation of soft tissue structures. However, it has not been widely assessed for measuring the scapholunate (SL) interval using dynamic stress views. This study aimed to determine whether ultrasound provides comparable measurements to radiography when evaluating the SL interval in healthy subjects. **Methods:** We analyzed 29 wrists from 29 individuals. Each wrist underwent dynamic stress maneuvers while the SL interval was measured with both radiography and US. The following views were obtained: neutral rotation, ulnar deviation, clenched fist, and a modified dynamic clenched fist view. The latter was performed by applying pressure on the head of the third metacarpal from distal to proximal. **Results:** The SL interval could be visualized in the clenched fist and ulnar deviation radiographic views, as well as in the modified dynamic clenched fist US view. Ultrasound in the neutral position produced measurements similar to those of traditional stress radiographs. The most pronounced changes in the SL interval occurred between neutral and dynamic views, whether assessed by radiography or US. There was no significant difference in the magnitude of interval change between the two imaging modalities. **Conclusions:** Ultrasound with dynamic stress maneuvers provided similar dynamic SL interval changes to stress radiographs in healthy individuals. A modified stress view using a slightly clenched fist and manual pressure on the third metacarpal head may offer hand surgeons a simple, real-time tool to noninvasively evaluate SL instability.

## 1. Introduction

Injuries to the scapholunate (SL) joint are the most frequent cause of carpal instability, although their diagnosis remains challenging for the hand surgeon [1]. The sequelae of these injuries account for considerable morbidity and, if left untreated, may lead to advanced scapholunate collapse and progressive deterioration of the carpus [2]. In a patient with pain around the SL complex, dynamic scapholunate instability (SLI) is present when a 3 mm widening of the SL interval is seen on stress radiographs but not on neutral radiographs [3,4].

Although many dynamic stress maneuver techniques have been described in the literature [5,6,7,8], no consensus has been reached on the optimal imaging for diagnosing this spectrum of lesions [9]. In 2021, an expert group suggested that standardized radiographs, radiographic stress views, dynamic fluoroscopy, magnetic resonance (MR) arthrography, and computed tomography (CT) arthrography are the most valuable and accurate imaging techniques for the work-up of SLI. Still, no gold standard has been defined [9].

Ultrasound (US) has been shown to be an effective tool in diagnosing SL ligament tears [10,11] but not in diagnosing dynamic SLI. Additionally, US is a useful tool in hand surgery due to its accessibility, portability, low cost, and noninvasive evaluation of soft tissue structures [12]. Only one study has evaluated US efficacy in assessing dynamic SLI [11]. However, this 2004 study only compared the SL intervals seen on US with arthroscopy, a more invasive imaging technique. In addition, advances in US technology were not considered in this study, which dates back over twenty years [11].

The main objective of the current study was to determine whether US provides comparable SL interval measurements to radiography in healthy subjects. The secondary objective was to determine the best dynamic US incidence for measuring a healthy SL joint space.

## 2. Materials and Methods

### 2.1. Study Design and Population

Patients were recruited prospectively from the orthopedic or emergency department for wrist examination in a tertiary academic hospital. These patients were initially admitted for unilateral hand wounds and were selected to participate in the study with their contralateral, healthy hand. Patients with a history of acute or chronic hand pain, hand injury, arthropathy, or radiographic abnormality were excluded. Informed consent was obtained from all patients. Twenty-nine participants were enrolled consecutively over a six-month period in 2023 (23 men, 6 women; mean age 38 years, range 19–60 years). Twenty right wrists were examined, of which 16 were the dominant hand.

### 2.2. Scapholunate Imaging Strategy

All patients were examined using the GE LOGIQ E9 sonographic unit (GE Healthcare, Milwaukee, WI, USA) and an ML 6 to 15 MHz high-resolution transducer (Supplemental Figure S1). The examinations were carried out by one investigator with specialized hand-surgery US experience (level III on Tang’s rating [13]).

**Figure 1:**
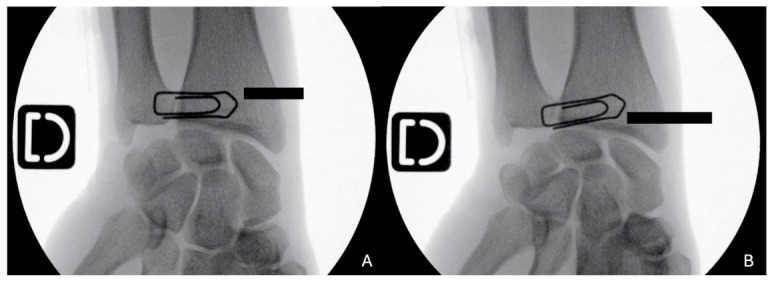
Radiographic images of the wrist after dynamic maneuvers; (**A**) Ulnar inclination at 30°, (**B**) Clenched fist pen incidence.

**Figure 2:**
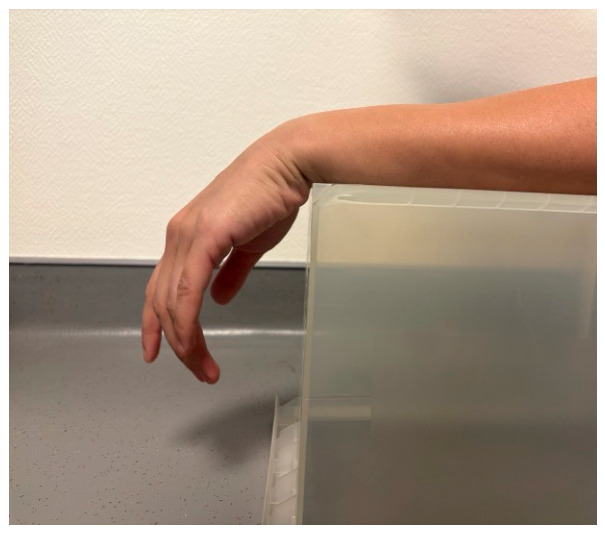
Forearm in neutral rotation and pronation on a wedge with the wrist slightly flexed at rest position.

**Figure 3:**
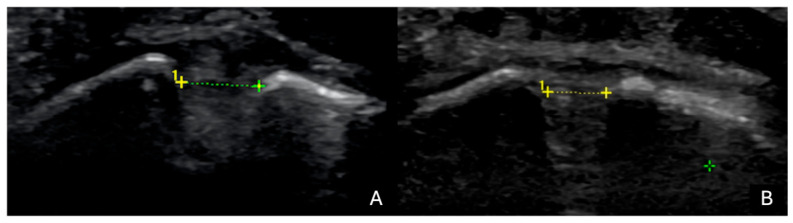
(**A**) Ultrasound images of the wrist after dynamic maneuvers with support on the head of the third metacarpal. (**B**) Ultrasound images of the wrist after dynamic maneuvers with the patient clenching their fist.

First, the scaphoid and lunate bones were visualized with the US transducer, and then the intervening dorsal portion of the SL interosseous ligament was identified. Dynamic US examinations were then performed by visualizing the SL ligament with the transducer applied dorsally while progressively flexing the wrist with a clenched fist. The forearm was positioned in pronation on a padded support, and the wrist was placed in slight flexion on a wedge to optimize dorsal visualization of the SL interval. The transducer was applied in a strictly transverse plane and adjusted until the dorsal cortices of the proximal pole of both the scaphoid and the lunate were simultaneously visualized in the same plane. For dynamic assessment, at least three loading cycles were performed per maneuver; the frame selected for measurement corresponded to the image showing maximal visible dorsal SL separation while maintaining sharp delineation of both cortical borders in the same plane. The SL interval was measured using the built-in electronic calipers as the distance between the dorsal cortical margins of the scaphoid and lunate. An OEC C-arm 9900 (GE Healthcare, Milwaukee, WI, USA) was used to obtain fluoroscopic images, and a standard radiographic marker, 1 cm in length, was used for calibration. The following specific dynamic stress views were tested on each patient:

− Posteroanterior neutral radiograph (PA-N XR): taken with the forearm in neutral rotation and pronation, the wrist in a neutral position, and the third metacarpal aligned with the longitudinal axis of the radius.
− Posteroanterior ulnar deviation (PA-UD XR): taken with the forearm in neutral rotation and pronation, the wrist in 30° of ulnar deviation and a neutral position, and the third metacarpal aligned with the longitudinal axis of the radius (Figure 1A).
− Posteroanterior clenched fist in ulnar deviation (PA-CF XR): taken with the forearm in neutral rotation and pronation, the wrist is in 30° of ulnar deviation in a neutral position with the fourth and fifth metacarpal heads flat on the cassette. The patient held a pencil, and it was positioned parallel to the cassette (Figure 1B) [5].

− At-rest flexed wrist ultrasound examinations (N-US): taken with the forearm in neutral rotation and pronation on a wedge with the wrist slightly flexed at rest position (Figure 2).
− Third metacarpal head push dynamic ultrasound examinations (M3-US): taken with the forearm in neutral rotation and pronation on a wedge with the wrist flexed and the fist slightly clenched. The examiner dynamically pushed on the head of the third metacarpal distally to proximally in the capitate’s axis, aiming to open the SL interval (Figure 3A).
− Clenched fist and flexed wrist dynamic ultrasound examinations (CF-US): taken with the forearm in neutral rotation and pronation on a wedge with the wrist flexed and the fist clenched at maximum level (Figure 3B).

### 2.3. Measurements and Statistical Analysis

As there is no consensus on the optimal technique for measuring SL intervals, it was calculated using fluoroscopy by measuring the distance between the scaphoid and lunate surfaces of the SL joint on the proximal aspect of the proximal carpal row. Dynamic US examinations demonstrated an echogenic fibrillar structure, and the gap was measured as the distance between the scaphoid and lunate borders of the SL joint on the dorsal part with the transducer transversely inclined. Two independent observers blinded to data from the other technique performed US and radiographic measurements. We also systematically calculated the inclination of the US transducer when the SL space was detected and measured at rest. We made two independent measurements of the SL interval and calculated the mean value. The association between transducer inclination and SL interval was assessed using linear regression (R^2^ reported). As the same participants underwent all six imaging conditions, pairwise comparisons between views were performed using the Wilcoxon signed-rank test, a non-parametric paired test appropriate for repeated measurements within the same subject. The significance threshold was set at α = 0.05. No correction for multiple comparisons was applied. Statistical analysis was conducted using JASP (version 0.19, Amsterdam, The Netherlands). A post-hoc sensitivity analysis indicated that with *n* = 29, the study had sufficient power (1 − β ≈ 0.80, α = 0.05, two-tailed) to detect a mean between-view difference of approximately 0.5 mm in the SL interval (SD ≈ 0.6 mm), which is consistent with the smallest difference observed in our data. The absence of a formal a priori power calculation is acknowledged as a limitation.

### 2.4. Ethical Approval

This study was approved by the local Institutional Review Board (IORG0012275—IULS, University Institute for Locomotion and Sport; IRB00014528—ULS, University Institute for Locomotion and Sport). Approval was granted for Research Project No. IRB00014528_2025_30: “Comparison of Radiographic and Ultrasonic Imaging Techniques for Assessing the Scapholunate Interval in Healthy Individuals.”

## 3. Results

Twenty-nine healthy wrists were examined in 29 patients (23 men; mean age, 38 years; range, 19–60 years; 20 right wrists).

**Figure 4:**
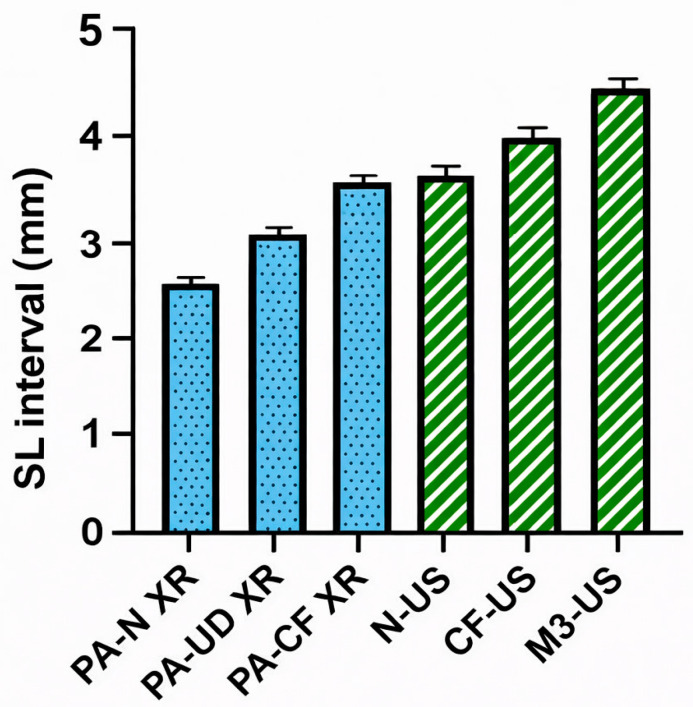
Average SL intervals (in mm) for the different radiographic and ultrasound images taken under stress.

Among the radiographic views, the PA-CF XR view provided higher mean SL interval measurements. Regarding US examination, the highest mean SL interval was achieved with the M3-US view (Table 1 and Table 2). The mean values of the SL interval gap for each dynamic stress view are reported in Figure 4.

Comparing radiographic views, PA-CF XR produced significantly greater SL interval measurements than PA-UD XR, whereas the PA-N XR view showed the lowest SL interval of all the radiographic views evaluated. Interestingly, the highest difference in SL interval values was observed between the PA-N XR view and the PA-CF XR view.

Comparing US views, the N-US view provided significantly lower SL interval values than the other two US views. Between the US dynamic views, M3-US provided higher SL interval measurements than CF-US, and this was the highest difference observed among the US views.

When comparing US and radiographic views directly, N-US produced measurements similar to those of PA-UD XR (Δ = 0.25 mm; *p* = 0.105) and PA-CF XR (Δ = 0.30 mm; *p* = 0.060), indicating that the resting ultrasound is comparable to the most commonly used radiographic stress views. Among all six views assessed, M3-US produced the highest mean SL interval overall, exceeding that of PA-CF XR and all other views regardless of imaging modality. The magnitude of SL interval change from neutral to the best dynamic maneuver was similar between modalities (PA-N XR to PA-CF XR: Δ = +1.16 mm versus N-US to M3-US: Δ = +0.99 mm; Δ = 0.17 mm; *p* = 0.180), suggesting that ultrasound dynamic assessment provides a comparable degree of SL interval opening to standard radiographic stress views. However, these direct cross-modality comparisons should be interpreted with caution, as the US and radiographic maneuvers are not strictly equivalent.

**Figure 5:**
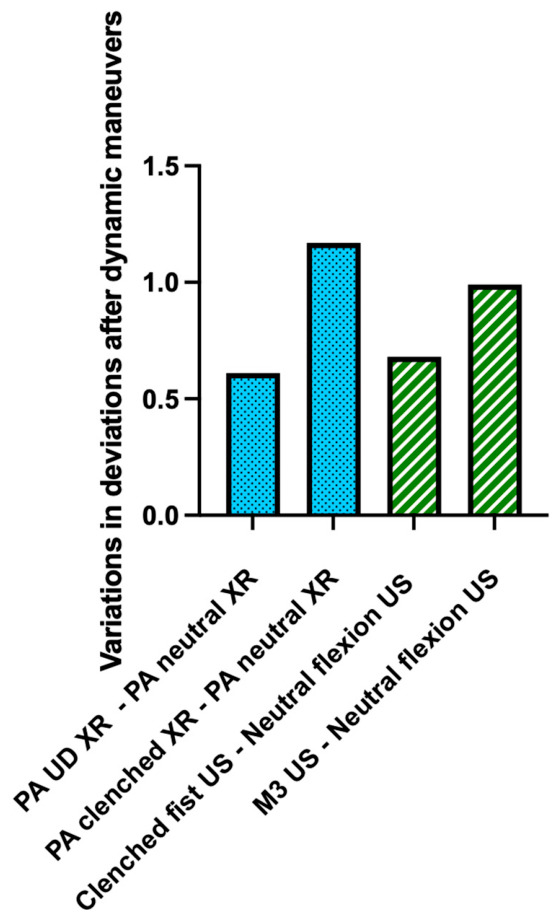
Variations in SL intervals (in mm) measured between neutral, radiographic, or ultrasound positions and positions under constraint.

The SL interval changes between neutral and dynamic views are shown in Figure 5.

The posteroanterior neutral radiograph was less efficient than all other stress views for SL interval widening visualization (*p* < 0.05). PA-CF XR was better than PA-UD XR for visualizing the SL gap (Δ = 0.55 mm; *p* = 0.009). N-US was similar to PA-UD XR (Δ = 0.25 mm; *p* = 0.105) and PA-CF XR (Δ = 0.30 mm; *p* = 0.06) but was less successful than both CF-US (Δ = 0.66 mm; *p* < 0.001) and M3-US (Δ = 0.99 mm; *p* < 0.001) for visualizing the SL interval. The most significant difference was observed between PA-N XR and PA-CF XR (Δ = +1.16 mm; range: 0.1–2.9 mm), but this difference was similar to that between M3-US and N-US (Δ = + 0.99 mm; range: −0.1–1.9 mm) (Δ = 0.17 mm; *p* = 0.18). Variations in the SL intervals from PA-N XR to PA-CF XR were greater than those from N-US to CF-US (Δ = +0.66 mm; range: −0.1–1.4 mm) (Δ = 0.50 mm; *p* = 0.002). The SL interval changes between neutral and dynamic views are shown in Figure 5.

The mean range of ultrasound transducer flexion at rest was 47° (33–70°), and no statistically significant association was found between the range of flexion and the SL interval (R^2^ = 0.07; *p* > 0.05).

## 4. Discussion

The findings from this study demonstrate that dynamic US examination provides similar results compared to dynamic stress radiographs in healthy patients. The M3-US view, achieved by pushing the head of the third metacarpal, provided the best results regarding the SL interval, although absolute values had a low clinical significance. Overall, dynamic maneuvers significantly increased the SL interval compared to rest in both modalities. The magnitude of interval change was greatest for PA-CF XR within radiography and for M3-US within ultrasound, supporting the conclusion that M3-US is the most effective ultrasound maneuver for SL interval assessment. Consequently, ultrasound examination by pushing the head of the third metacarpal appears to be the most effective modality assessed in this study for measuring the SL interval in healthy patients. We did not identify any preferential transducer inclination for better SL space detection at rest.

In the literature, radiographic views with ulnar deviation or clenched pencil views are considered the most effective in visualizing dynamic SLI [6]. A cadaveric study by Lee et al. assessed eight different stress views and showed that the pencil view with a clenched fist was the most effective view to display SL widening [5]. The study by Lee et al. also found that a tilt of 30° for ulnar deviation is most effective in demonstrating SL widening and that the SL interval decreases above this threshold [5]. The clenched pencil view helps provide a bilateral comparative assessment of the SL interval. On standard radiographs in the neutral position, a threshold of 3 mm is commonly considered for the diagnosis of SLI [14]. However, differentials are more variable in the case of dynamic SLI and may differ from 2 to 5 mm according to the various techniques for measuring the SL interval. Dornberger et al. found that Stecher’s projection radiographs with a closed fist and ulnar deviation had the most accurate sensitivity and specificity for diagnosing SL complex tears, with thresholds of 3 or 3.7 mm [15].

Previous research has described several techniques to obtain the largest SL interval when the wrist has a physiologic load [4,7,16], yet there is currently no established gold standard. Real-time, three-dimensional magnetic resonance imaging (MRI) also allows detection of subtle SL interval variations, which may improve the accuracy for the diagnosis of dynamic SLI [17]. However, it is an expensive imaging modality that is often difficult to access. There may also be limitations with standards and dynamic radiographs, since partially healed, acute SL ligament tears may be associated with either a negative result or a delay between clinical assessment and imaging. Artifacts of chronic inflammation may lead to the same errors [18]. Therefore, US may help to quickly identify injuries to the extrinsic and intrinsic carpal ligaments [10,19]. Moreover, this examination can be performed by the trained surgeon, thereby simplifying the diagnostic circuit for the patient [20,21]. Ultrasound can also predict the diagnosis of tears of the dorsal band of the SL ligament with a sensitivity of 46–100% and a specificity of 92–100% [22]. Gondim Teixera et al. found that the dorsal SL ligament was not visible in 15% of cases without indication of an injury, whereas the proximal component of the SL ligament was seen in all cases [23]. Similarly, we did not clearly visualize the dorsal part of the SL ligament in nine cases (31%). This seems to be due to the quality of the ultrasound examination.

If US provides comparable results to radiography in measuring the SL interval, it also carries another advantage. Indeed, US enables the correct analysis of structures invisible on radiographs, such as the dorsal capsulo-scapholunate septum [24] or the dorsal part of the interosseous ligament. The direct observation of the SL ligament by US can help analyze signs of disruption of the dorsal aspect of the SL ligament [11,21], such as an absence of continuous hyperechoic ligament fibers between the scaphoid and lunate bones or a hypoechogenic discontinuity [25]. Dynamic ultrasound examinations of the dorsal portion of the SL ligament have been described with a clenched fist to detect dynamic instability. Dao et al. reported a sensitivity of 46% and a specificity of 100% [11]; the low sensitivity could be due to arthroscopy being the gold standard or to pre-dynamic instability, which is not detected by conventional or dynamic radiographs [3]. Another study showed that a tightly squeezed clenched fist, without radial or ulnar deviation, was a reliable method for assessing the SL ligament in healthy patients; however, there was no significant difference in the SL ligament interval length between the squeeze test and US performed at rest [26].

Besides assessing the SL interval, the quantification of dorsal subluxation of the scaphoid in relation to the lunate using US seems to be an effective method for diagnosing SLI. Indeed, in 2021, a Swiss team showed that the dorsal subluxation of the scaphoid can be accurately measured on US with excellent interobserver reliability between four experienced hand surgeons using a standardized 3D test model [27]. This was confirmed by a clinical trial published in 2024 on 20 patients with SLI in whom US was used during the Watson test preoperatively [28].

The current study has several limitations. First, US examination was performed by one investigator, which did not allow for inter-examiner reliability. However, US and radiographic measurements were performed by two independent observers blinded to data from the other technique.

Second, there was no comparison group of patients with dynamic SLI. Third, the study has a small sample size, which limits the generalizability of the results. Larger comparative studies on patients with SLI are needed to confirm this study’s results and enhance the clinical relevance of dynamic stress US examination. Moreover, wrist arthroscopy, considered the gold standard for evaluating ligamentous instability, should be performed on patients with SLI and assessed by US to determine the efficiency of dynamic stress maneuvers in symptomatic patients. Finally, the maneuvers compared between US and radiography are not strictly equivalent, which limits the validity of direct comparisons between the two modalities. However, pushing on the third metacarpal during radiography would be difficult to translate into clinical practice. Therefore, we consider this view to be a specificity that ultrasound can provide.

## 5. Conclusions

This study’s findings suggest that US examination with dynamic stress maneuvers appears clinically comparable to stress radiographs for viewing SL intervals in healthy patients. A modified stress view with a fist slightly clenched while pressure is applied to the head of the third metacarpal seems to be the best view for SL interval assessment. For a clinician assessing SL complex injuries, US could offer a valuable method for obtaining a SL stress view.

## Figures and Tables

**Table 1:** Mean scapholunate interval values by view (*n* = 29 wrists).

View	Modality	Mean SL Interval (mm)	Range (mm)
PA-N XR	Radiography	2.01	1.1–3.2
PA-UD XR	Radiography	2.62	1.5–4.0
PA-CF XR	Radiography	3.17	1.8–4.6
N-US	Ultrasound	2.87	1.5–4.2
CF-US	Ultrasound	3.53	1.7–4.9
M3-US	Ultrasound	3.86	2.1–5.5

The values are means with ranges in parentheses. PA-N XR = posteroanterior neutral radiograph; PA-UD XR = posteroanterior ulnar deviation; PA-CF XR = posteroanterior clenched fist; N-US = at-rest ultrasound; CF-US = clenched fist ultrasound; M3-US = third metacarpal head push ultrasound.

**Table 2:** Pairwise comparisons of scapholunate interval measurements between views.

Comparison	Mean Difference (mm)	*p*-Value
Within radiography		
PA-N XR vs. PA-UD XR	0.61	0.031
PA-N XR vs. PA-CF XR	1.16 (0.1–2.9)	<0.001
PA-UD XR vs. PA-CF XR	0.55	0.009
Within ultrasound		
N-US vs. CF-US	0.66 (−0.1–1.4)	<0.001
N-US vs. M3-US	0.99 (−0.1–1.9)	<0.001
CF-US vs. M3-US	0.33 (0.0–0.8)	0.044
Cross-modality comparisons		
N-US vs. PA-UD XR	0.25	0.105 (NS)
N-US vs. PA-CF XR	0.30	0.060 (NS)
ΔPA-CF XR − PA-N XR vs. ΔM3-US − N-US	0.17	0.180 (NS)
ΔPA-CF XR − PA-N XR vs. ΔCF-US − N-US	0.50	0.002

NS = not significant. Δ = mean difference in SL interval. Wilcoxon signed-rank tests (α = 0.05). No correction for multiple comparisons applied.

## Data Availability

The original contributions presented in this study are included in the article. Further inquiries can be directed to the corresponding author.

## Supplementary Materials

The following supporting information can be downloaded at: https://www.mdpi.com/article/10.3390/jcm15114250/s1, Figure S1: Illustration of ultrasound examination procedures.

## Abbreviations

The following abbreviations are used in this manuscript:

STROBE	Strengthening the Reporting of Observational Studies in Epidemiology
CF-US	Clenched fist and flexed wrist dynamic ultrasound examinations
CT	Computed tomography
M3-US	Third metacarpal head push dynamic ultrasound
MR	Magnetic resonance
MRI	Magnetic resonance imaging
N-US	At-rest flexed wrist ultrasound examination
PA-CF XR	Posteroanterior clenched fist in ulnar deviation radiograph
PA-N XR	Posteroanterior neutral radiograph
PA-UD XR	Posteroanterior ulnar deviation radiograph
R2	Coefficient of determination
SL	Scapholunate
SLI	Scapholunate instability
JASP	Statistical software used for all analyses
US	Ultrasound
