# Supplementary figures and images for: Comparison of Radiographic and Ultrasound Imaging Techniques for Assessing the Scapholunate Interval in Healthy Individuals
Source: J Clin Med. 2026 May 31;15(11):4250. doi: 10.3390/jcm15114250 (PMC13258590; doi:10.3390/jcm15114250)

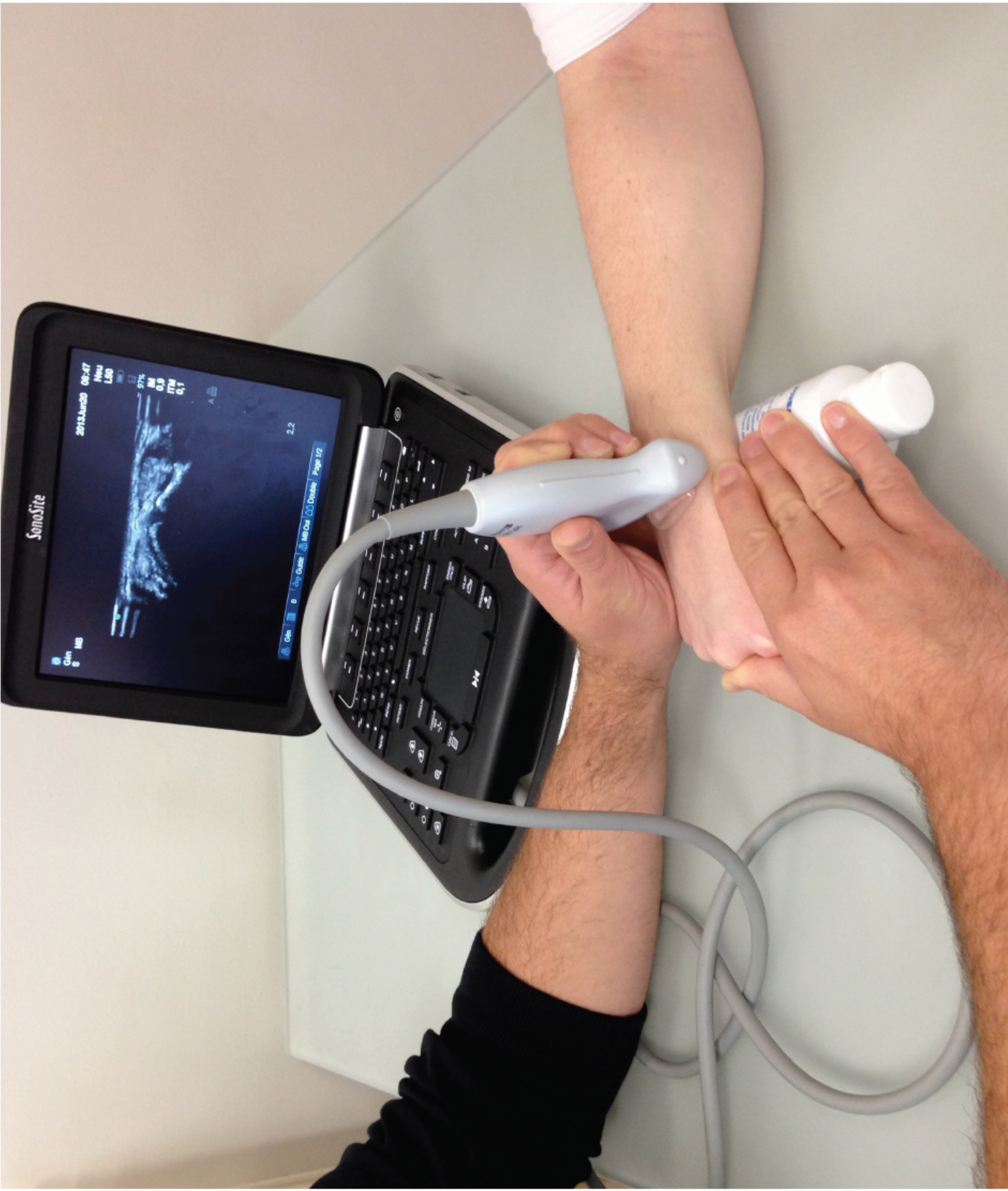

Supplement: Supplementary file 1 [file jcm-15-04250-s001.zip › jcm-4209038-Supplementary Figure S1.pdf]
